# Outcomes of thoracic endovascular aortic repair for blunt thoracic aortic injury with emphasis on short proximal sealing zones ≤ 15 mm

**DOI:** 10.1186/s42155-026-00717-4

**Published:** 2026-06-12

**Authors:** Yonghun Kim, Yohan Kwon, Jeong Min Lee, Jee Hyun Baek, Jinoo Kim, Je Hwan Won, Tae Won Choi

**Affiliations:** 1 Department of Radiology, Ajou University School of Medicine, Suwon, South Korea; 2 Department of Radiology, Korea University Anam Hospital, Seoul, South Korea; 3 Seoul 88 Clinic, Suwon, South Korea

**Keywords:** Trauma, Blunt thoracic aortic injury, Thoracic endovascular aortic repair, Stent graft, Short proximal sealing zone

## Abstract

**Purpose:**

Blunt thoracic aortic injury (BTAI) is the second leading cause of mortality in patients with trauma, closely following head injuries, which remain a significant concern in trauma care. Thoracic endovascular aortic repair (TEVAR) is the preferred treatment for BTAI; however, insufficient proximal sealing zone length poses a significant challenge for successful TEVAR. This study aimed to investigate the outcomes of TEVAR in patients with BTAI, including those with short proximal sealing zones (≤ 15 mm).

**Materials and methods:**

This retrospective study included 52 consecutive patients who underwent TEVAR for BTAI in the authors’ institution between January 2018 and December 2023. Patient demographics, BTAI grade, adverse events, and endoleak were assessed. Proximal sealing zone length was measured on pre-procedural computed tomography (CT). Subgroup analysis was used to compare outcomes between patients with proximal sealing zones ≤ 15 and > 15 mm.

**Results:**

The average injury severity score was 44.1 ± 15.8, with 88.5% of patients sustaining grade 3 BTAI. One technical failure occurred owing to an acute aortic arch angle. A total of 29 patients had proximal sealing zones ≤ 15 mm, with 5 of them undergoing left subclavian artery coverage. Endoleak occurred in three patients (5.9%), resulting in the death of one patient. The remaining two patients had suspected type 4 endoleaks that resolved on follow-up CT. No significant differences were observed in aortic-related mortality and endoleak between the two groups.

**Conclusions:**

TEVAR is an effective treatment option for BTAI, even in patients with short proximal sealing zones ≤ 15 mm.

## Introduction

Blunt thoracic aortic injury (BTAI) is the second leading cause of mortality in patients with trauma, closely following head injuries, which remain a significant concern in trauma care [1, 2]. According to the US National Trauma Data Bank for the years 2000–2005, 24% of patients with BTAI either died upon arrival or during triage. Among those who survived triage but did not receive aortic repair, mortality was as high as 68% [3].

Thoracic endovascular aortic repair (TEVAR) is the preferred treatment for BTAI, as recommended by the Society for Vascular Surgery [4] and European Society of Cardiology [5]. However, insufficient proximal sealing zone length poses a significant challenge for successful TEVAR, since aortic injuries often occur at the isthmus, located 10–20 mm from the origin of the left subclavian artery (LSA) [6–8]. In patients with short proximal sealing zones, stent-graft coverage of the LSA is considered necessary to secure a sufficient proximal sealing zone length and seal the aortic injury site [7, 9, 10]. However, this approach carries risks of posterior circulation stroke, left arm ischemia, and spinal cord injury [10–12].

The concept of a minimum proximal seal length of 20 mm, originally established for aneurysmal disease management, has been applied in BTAI [8]. However, aortic aneurysms typically affect aortas with severe underlying atherosclerosis, whereas aortas in BTAI are often normal, necessitating a shorter proximal sealing zone length in TEVAR [7, 8]. Nevertheless, the ideal length of the proximal seal for minimizing early and late complications remains debatable, with recommendations ranging from 15 to 30 mm [7, 8, 13–16].

Therefore, this study aimed to investigate the outcomes of TEVAR in patients with BTAI at a level 1 trauma center between those with proximal sealing zones ≤ 15 and > 15 mm.

## Materials and methods

### Patient population

This retrospective study was approved by the Institutional Review Board of the authors’ institution, and the need for written informed consent was waived owing to the study design. A total of 52 patients with BTAI, who were suitable for TEVAR based on the treatment protocol [17] between January 2018 and December 2023, were included in the study.

### Pre-procedural computed tomography angiography

All patients underwent pre-procedural computed tomography (CT) to identify the lesions before treatment. CT scans were acquired using a 16-channel scanner (SOMATOM Sensation 16, Siemens Healthineers, Forchheim, Germany) with the following imaging parameters: peak voltage, 120 kVp; tube current, 100–280 mAs; slice thickness, 2.5 mm; reconstruction interval, 2.5 mm; and beam collimation, 0.625 mm. Following pre-contrast scans, 90 mL of a nonionic contrast medium (100 mL of Pamiray 370, Dongkook Pharmaceutical, Seoul, South Korea) was injected intravenously at a flow rate of 2.5 mL/s using an automatic power injector, and enhanced images were obtained 45 s post-injection.

### TEVAR procedure

The femoral artery was accessed percutaneously using the pre-close technique whenever possible [18], whereas a surgical cut-down approach was performed in complex cases, such as severe vessel wall calcification. TEVAR was performed under local anesthesia or deep sedation using propofol, fentanyl, dexmedetomidine, and vecuronium, as deemed appropriate. Initial aortography was performed using a 5-Fr pigtail catheter (Royal Flush Plus Pigtail Catheter, Cook Medical, Bloomington, IN, USA) to assess aortic injury, sealing zone, and branch vessel anatomy. A thoracic stent-graft was then inserted over the Lunderquist extra-stiff guidewire (Cook Medical) under fluoroscopic guidance. Stent-graft size was determined to be 10–30% larger than the proximal aortic diameter, considering the patient's hemodynamic status [19–21]. Because aortic caliber may be underestimated in hemodynamically unstable patients, 10–20% oversizing was used in stable patients, whereas 20–30% oversizing was applied in unstable patients. Post-procedural angiography was conducted to confirm adequate injury site coverage. A molding balloon was used only in cases with immediate type I endoleaks.

### Follow-up CT angiography

To evaluate for endoleaks and LSA status, follow-up CT scans were performed in all patients, excluding those with an expired status or TEVAR failure. Scans were conducted within 2 weeks, and then at 1, 6, and 12 months post-intervention, and annually thereafter.

The follow-up CT protocol involved obtaining unenhanced CT images and two-phased contrast CT images with intravenous administration of nonionic contrast medium (150 mL of Xenetix 300, Guerbet SA, Villepinte, France) at a dose of 2.0 mL/kg (up to 140 mL). Arterial and delayed phase images were acquired at 8 and 60 s, respectively, after the attenuation in the abdominal aorta reached 100 Hounsfield units.

### Data analysis

Patient characteristics and procedural details were obtained from the electronic medical records at the authors’ institution. Technical success was defined as successful stent-graft deployment without immediate endoleak confirmed using completion angiography. Clinical outcomes, including mortality, aortic-related mortality, adverse events, endoleak, length of hospital stay, and time in intensive care unit, were obtained from electronic medical records and follow-up CT angiography. Adverse events were evaluated based on the modified Society of Interventional Radiology adverse event classification system [22]. Aortic-related mortality was defined as death directly attributable to the index thoracic aortic pathology or its treatment (TEVAR), including rupture, malperfusion, or procedure-related complications. Deaths not attributable to thoracic aortic disease and those with indeterminate causes were classified as non–aortic-related mortality.

**Fig. 1:**
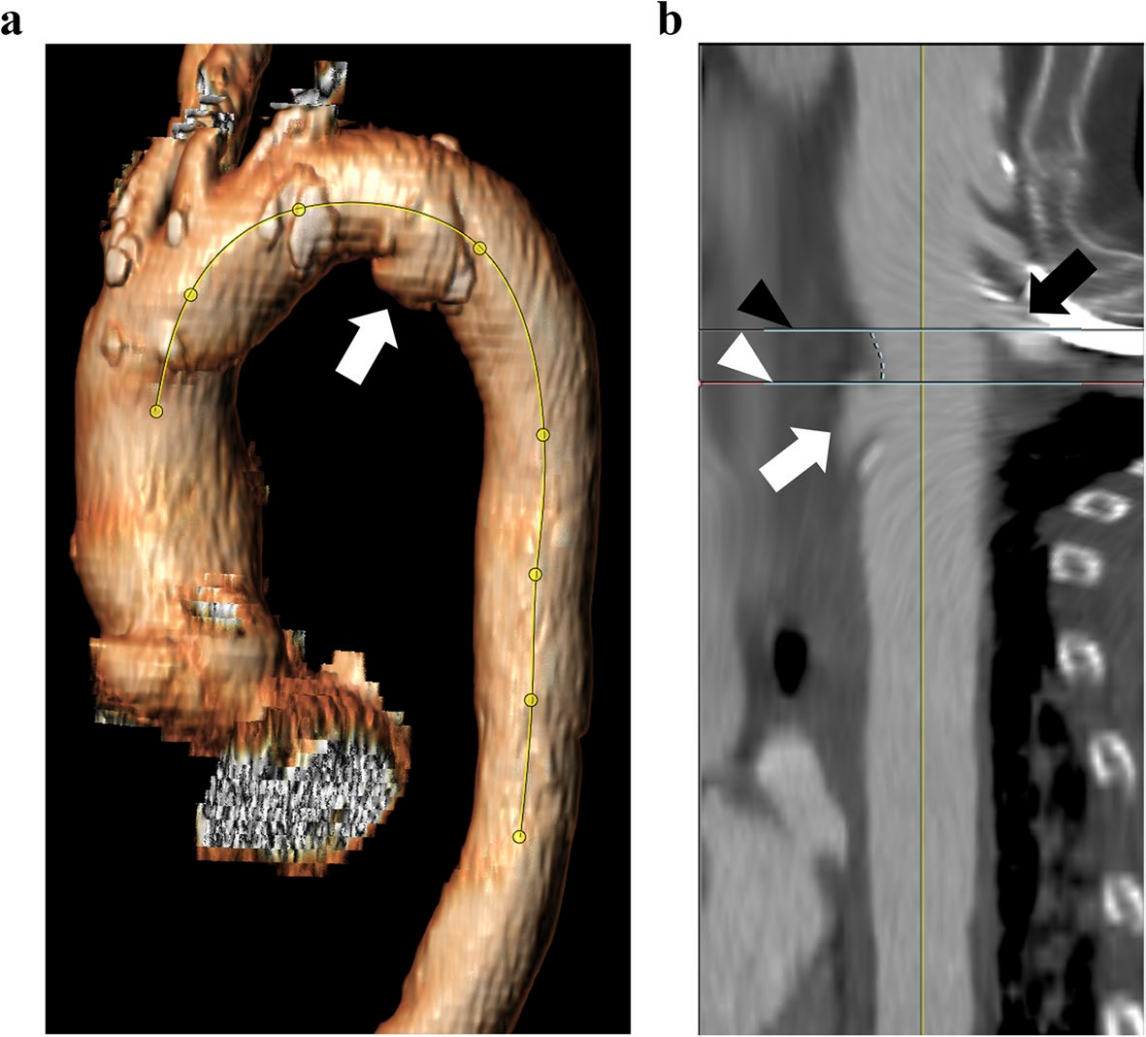
Measurement of the proximal sealing zone length using the 3mensio Vascular software (Pie Medical Imaging). **A** The centerline of the thoracic aorta is determined. The aortic injury site is located on the lesser curvature side (Arrow). **B** On the stretched vessel view, the proximal left subclavian artery (black arrow) and aortic injury site (white arrow) are noted. To calculate the centerline length, two planes perpendicular to the centerline (blue lines) are positioned at the left subclavian artery origin (black arrowhead) and aortic injury site (white arrowhead)

The 3Mensio Vascular software (Pie Medical Imaging, Bilthoven, the Netherlands) was used to reconstruct CT images and measure the proximal sealing zone length (Fig. 1). Briefly, one reviewer determined the centerline of the thoracic aorta and positioned two perpendicular planes at the aortic injury site and LSA origin. The centerline length between the two planes was then calculated using the software. To assess interobserver variability in proximal sealing zone measurement, a second reviewer independently measured the proximal sealing zone length using the same method.

Subgroup analysis compared the outcomes of patients with proximal sealing zones ≤ 15 and > 15 mm.

### Statistical analysis

Categorical variables were compared using the chi-squared test (if expected cell size < 5) or Fisher’s exact test, whereas continuous variables were compared using Student’s t-test. The intraclass correlation coefficient (ICC) was calculated to assess interobserver variability in proximal sealing zone length measurements. All statistical analyses were performed using the SPSS statistical software version 25 (IBM, Armonk, NY, USA) and MedCalc statistical software version 20.1 (MedCalc Software, Ostend, Belgium), and statistical significance was set at P < 0.05.

## Results

### Patient characteristics

Patient characteristics are summarized in Table 1. The mean age was 46 ± 16 years (range 15–79 years), with 42 (80.8%) being male. The mean injury severity score was 44.1 ± 15.8 (range 17–75). Of the enrolled patients, 88.5% had grade 3 BTAI. Notably, one patient presented with aortic dissection only, which was classified as grade 2 [23, 24]. In 37 (71.2%) patients, the injury involved the lesser curvature of the aortic arch, whereas in 3 (5.8%) patients it involved the greater curvature. In the remaining 12 (23.1%) patients, the injury was multifocal; however, the most proximal site relevant to the stent-graft sealing zone was on the lesser curvature in all 12 patients. Regarding procedure timing, 45 patients (86.5%) underwent TEVAR within 24 h of admission.

**Table 1:** Patient characteristics

Characteristics		Value
Age (years)		46 ± 16 [15–79]
Sex (male)		42 (80.8)
Injury Severity Score		44.1 ± 15.8 [17–75]
Mechanism of injury	Motor vehicle accident	17 (32.7)
	Pedestrian traffic accident	5 (9.6)
	In-car traffic accident	12 (23.1)
	Fall	14 (26.9)
	Cushing injury due to machine	4 (7.7)
BTAI grade	Grade 2	1 (1.9)
	Grade 3	46 (88.5)
	Grade 4	5 (9.6)
Aortic curvature at injury site	Lesser curvature	37 (71.2)
	Greater curvature	3 (5.8)
	Multifocal	12 (23.1)
Time to procedure	Early (< 24 h)	45 (86.5)
	Late (> 24 h)	7 (13.5)

^†^Continuous data are presented as means ± standard deviation [range]. Categorical data are presented as numbers (%)

*BTAI* = blunt thoracic aortic injury

### Endovascular repair

Procedural details are summarized in Table 2. Stent-graft placement was technically successful in 51 (98.1%) patients, with complete initial injury site exclusion. Technical failure was documented in one patient owing to the difficulty of stent-graft insertion into the acute aortic arch angle, necessitating open surgical repair.

**Table 2:** Procedural details

Characteristics		Value
Technical success		51 (98.1)
Percutaneous access		51 (98.1)
Length of proximal sealing zone (mm)		18.7 ± 13.9 [0–78]
Left subclavian artery coverage		6 (11.8)
Aortic diameter	Proximal (mm)	23.7 ± 4.1 [14.9–37.0]
	Distal (mm)	20.2 ± 4.1 [13.1–30.8]
Stent graft size	Diameter (mm)	30.1 ± 3.9 [22–44]
	Length (mm)	136.5 ± 27.0 [96–212]
Post-balloon		1 (2.0)
Procedure time (min)		48.2 ± 23.2 [27–186]

^†^Continuous data are presented as means ± standard deviation [range]

Categorical data are presented as numbers (%)

The mean proximal sealing zone length was 18.7 ± 13.9 mm, and the interobserver agreement for the measurement was excellent (ICC = 0.991, 95% CI 0.984–0.995). The LSA coverage was performed in six patients. Regarding stent-graft type, Talent and Valiant Captivia (Medtronic, Santa Rosa, CA, USA) was used in 43 (82.7%) patients, whereas Zenith TX2 and Zenith Alpha (Cook Medical) was used in 9 patients (17.3%).

### Clinical outcomes and adverse events

Clinical outcomes and adverse events are presented in Table 3. Excluding the three patients who expired, all remaining patients underwent follow-up CT scans within 2 weeks post-procedure. The mean follow-up period was 348.4 ± 363.3 days. Endoleak occurred in three patients, resulting in the death of one of the patients. This patient presented with grade 4 BTAI, with a proximal sealing zone length of 23 mm. TEVAR was performed using Talent and Valiant Captivia device (Medtronic). Post-procedural CT revealed the presence of an endoleak, leading to hemodynamic instability and eventually death. The two remaining patients, both treated with Talent and Valiant Captivia devices (Medtronic), were suspected to have type 4 endoleaks that resolved on follow-up.

**Table 3:** Clinical outcomes

Characteristics		Total	Proximal sealing zone length		P
			**≤ 15 mm**	**> 15 mm**	
Number		51	29	22	
Left subclavian artery coverage		6 (11.8)	5 (17.2)	1 (4.5)	.218
Injury Severity Score		44.1 ± 15.8 [17–75]	44.9 ± 16.0 [21–75]	43.0 ± 15.9 [17–75]	.675
In-hospital mortality		8 (15.7)	4 (13.8)	4 (18.2)	.713
Aortic-related mortality (thoracic)		1 (2.0)	0 (0.0)	1 (4.5)	.431
Non-aortic-related mortality (thoracic)	Total	7 (13.7)	4 (13.8)	3 (13.6)	1.000
	Brain injury	3 (5.9)	3 (10.3)	0 (0.0)	
	Abdominal aorta	1 (2.0)	0 (0.0)	1 (4.5)	
	Indeterminate (polytrauma)	3 (5.9)	1 (3.4)	2 (9.1)	
Endoleak		3 (5.9)	1 (3.4)	2 (9.1)	.571
Complication	Access site	3 (5.9)	2 (6.9)	1 (4.5)	1.000
	Left arm claudication	1 (2.0)	1 (3.4)	0 (0.0)	1.000
	Stroke	0 (0.0)	0 (0.0)	0 (0.0)	–
	Spinal cord ischemia	0 (0.0)	0 (0.0)	0 (0.0)	–
Reintervention		2 (3.9)	2 (6.9)	0 (0.0)	.500
Left subclavian artery bypass		1 (2.0)	1 (3.4)	0 (0.0)	1.000
Hospital stay (days)		32.9 ± 33.5 [1–210]	40.3 ± 39.9 [2–210]	23.0 ± 19.4 [1–91]	.067
ICU stay		13.8 ± 15.1 [1–84]	17.6 ± 18.4 [2–84]	8.5 ± 6.4 [1–22]	.016

^†^Continuous data are presented as means ± standard deviation [range]. Categorical data are presented as numbers (%)

Overall, in-hospital mortality rate was 15.7%. The causes of mortality are summarized in Tables 3 and 4. One patient died from aortic injury presenting primarily as abdominal aortic dissection with multiple dynamic and static occlusions, resulting in malperfusion of the abdominal organs and both iliac arteries. There was no evidence of injury at the aortic isthmus. TEVAR was performed for extension of the dissection into the thoracic aorta, and additional stent placement was performed for the iliac artery lesions. Despite these interventions, the patient eventually died from progressive multiorgan failure secondary to malperfusion. Three patients died due to brain injury. The remaining three patients had multiple traumatic injuries; although concomitant aortic injury was present, its contribution to mortality was indeterminate, and these deaths were classified as non–aortic-related mortality.

**Table 4:** Clinical outcomes in patients without LSA coverage

Characteristics		Total	Proximal sealing zone length		P
			**≤ 15 mm**	**> 15 mm**	
Number		45	24	21	
Injury Severity Score		43.3 ± 15.1 [17–75]	44.8 ± 15.7 [21–75]	41.5 ± 14.5 [17–75]	.463
In-hospital mortality		6 (13.3)	3 (12.5)	3 (14.3)	1.000
Aortic-related mortality(thoracic)		1 (2.2)	0 (0.0)	1 (4.8)	.467
Non-aortic-related mortality (thoracic)	Total	5 (11.1)	3 (12.5)	2 (9.5)	1.000
	Brain injury	2 (4.4)	2 (8.3)	0 (0.0)	
	Abdominal aorta	1 (2.2)	0 (0.0)	1 (4.8)	
	Indeterminate (polytrauma)	2 (4.4)	1 (4.2)	1 (4.8)	
Endoleak		3 (6.7)	1 (4.2)	2 (9.5)	.592
Complication	Access site	2 (4.4)	1 (4.2)	1 (4.8)	.923
	Left arm claudication	0 (0.0)	0 (0.0)	0 (0.0)	-
	Stroke	0 (0.0)	0 (0.0)	0 (0.0)	-
	Spinal cord ischemia	0 (0.0)	0 (0.0)	0 (0.0)	-
Reintervention		1 (2.2)	1 (4.2)	0 (0.0)	1.000
Hospital stay (days)		34.1 ± 35.2 [1–210]	42.9 ± 43.3 [2–210]	24.1 ± 19.2 [1–91]	.063
ICU stay		14.4 ± 15.7 [1–84]	19.3 ± 19.6 [2–84]	8.9 ± 6.3 [1–22]	.020

^†^Continuous data are presented as means ± standard deviation [range]. Categorical data are presented as numbers (%)

Four patients experienced adverse events: three with access site complications and one with left arm claudication. Among them, two patients with access site complications required additional procedures, such as balloon angioplasty, whereas the remaining two improved with conservative management.

Among the five patients who underwent TEVAR with LSA coverage without bypass surgery, only one experienced a minor adverse event, left arm claudication, which improved during follow-up. However, mild intermittent left arm paresthesia persisted during 4 years of follow-up. The remaining four patients had a mean follow-up duration of 346 days and remained asymptomatic during follow-up.

Our retrospective study included only one pediatric patient, a 15-year-old with multiple traumatic injuries from a fall. The decision to perform TEVAR was made through multidisciplinary discussion with the trauma team. The procedure was technically successful; however, an access site complication developed, requiring surgical repair. The patient recovered and was discharged 2 weeks later. Follow-up CT scans at 1 and 4 months demonstrated successful coverage of the injury site without complications. In addition, no procedure-related symptoms or complications were observed during 4 years of follow-up.

### Clinical outcomes in patients with proximal sealing zones ≤ 15 mm

Comparisons of clinical outcomes between patients with proximal sealing zones ≤ 15 and > 15 mm are presented in Table 3. Of the enrolled patients, 29 had a proximal sealing zone ≤ 15 mm, with 5 requiring LSA coverage. No occurrences of aortic-related mortality were reported, and one case of endoleak occurred in this group. Furthermore, no significant differences in aortic-related mortality and endoleak were observed between the two groups (*P* > 0.05). Subgroup analysis of 45 patients without LSA coverage is summarized in Table 4.

## Discussion

This study demonstrated a high technical success rate of 98.1% for TEVAR in patients with BTAI, aligning with previous studies reporting rates from 96 to 100% [7, 8]. Among the eight cases of in-hospital mortality, only one case of aortic-related mortality (2%) was observed. This is consistent with prior reports on TEVAR showing aortic-related mortality rates of 2–7% [7, 12, 14, 15, 25]. Similarly, the incidence of endoleak in this study was 5.9%, falling within the range of 0–13.6% reported in previous studies [7, 8, 24, 26]. However, the mean follow-up duration was 11.6 months, which was slightly shorter than follow-up periods of 17–24 months in other studies [8, 24, 25]. This difference is likely owing to the more recent patient population in this study, who were selected to minimize device variation and reflect the use of contemporary devices.

Aortic injury often occurs at the isthmus (10–20 mm from the LSA), resulting in insufficient proximal sealing zone lengths [8] While the cutoff for a short proximal sealing zone length varies among studies, with some defining this at 20 mm [7, 8, 24] or 15 mm [27], these lengths are derived from the thoracic aortic aneurysm treatment rather than BTAI. Therefore, a debate regarding its sufficient length in BTAI is ongoing. The aortas in most patients with BTAI are pathologically normal compared to those in other aneurysmal diseases requiring TEVAR [7, 8, 28]. Moreover, TEVAR can be performed without LSA coverage in cases with short proximal sealing zones < 20 mm. Skripochnik et al. [8] reported that a shorter sealing zone of 10–20 mm may be safe and feasible for maintaining a patent LSA. Likewise, Pu et al. [25] stated that a proximal sealing zone < 15 mm was not an absolute contraindication for TEVAR. Given these findings, our study designated 15 mm as the cutoff for a short proximal sealing zone, revealing no significant differences in mortality and adverse events between the two groups. Additionally, the outcomes of the short proximal sealing zone group of this study demonstrated comparable outcomes with previous literature in terms of aortic-related mortality (0% vs. 0%) and endoleak incidence (3.4% vs. 0–12.2%) [7, 8, 24].

Various complications have been associated with LSA coverage, including stroke (2.4%) and left arm ischemia (3–15%) [10–12]. Among the six patients who underwent TEVAR with LSA coverage in our study, one experienced a minor adverse event, left arm claudication. Although pre-procedural revascularization, such as bypass surgery, has been recommended for LSA coverage, specifically in emergency TEVAR, it is often not feasible. In addition, alternative carotid-subclavian bypass surgery may result in adverse events, such as phrenic nerve injury, recurrent laryngeal nerve palsy, axillary nerve palsy, and neck hematoma [29]. In young patients, carotid-subclavian bypass surgery has also been criticized owing to the lack of long-term follow-up data on integrity and durability [30]. Therefore, considering the comparable outcomes in patients with short proximal sealing zones ≤ 15 mm, TEVAR without LSA coverage may be viable to reduce associated morbidities. Further studies, including randomized controlled trials with large populations, are warranted to validate these findings.

Recently, alternative strategies to mitigate complications related to LSA coverage, including fenestrated TEVAR, chimney techniques, and single-branched devices such as the Gore TAG Thoracic Branch Endoprosthesis (W. L. Gore & Associates, Flagstaff, AZ, USA) and MicroPort Cratos (MicroPort Medical, Shanghai, China), have been increasingly investigated [31–33]. However, single-branched devices may not be available depending on the region or clinical setting, as in our study. In addition, fenestrated or chimney techniques were not routinely applied because most cases involved urgent aortic injury, in which procedural time and device availability were limited. Instead, a debranching strategy with subclavian artery bypass was considered after TEVAR when necessary.

In our center, 86.5% of patients underwent TEVAR within 24 h of admission. The optimal timing of TEVAR for BTAI remains a topic of debate [2]. While some studies report delayed TEVAR after 24 h reduces overall mortality compared to early TEVAR [34–36], others show no significant difference between the two approaches [37, 38]. One potential explanation for the benefit of delayed TEVAR is the possibility of overlooking accompanying injuries in patients with multiple traumas who undergo early TEVAR [34]. Despite this, further research is needed to clarify optimal timing, and decisions regarding TEVAR timing should be based on individual and trauma center factors.

Different methods of measuring the proximal sealing zone length have been proposed, including the length along the inner curve and the length on the centerline [8, 16, 39]. However, in our experience, the proximal sealing zone length along the inner curve drawn by the software was occasionally not on the lesser curvature of the aortic arch because of its three-dimensionality. In addition, the BTAI site was not on the lesser curvature side and more adjacent to the greater curvature side in rare cases. Therefore, we reported the length of the proximal sealing zone as that along the centerline, similar to how it was defined in several previous studies [8, 16]. However, caution should be exercised when comparing results across studies owing to potential differences in measurement methods [39].

This study also has some limitations. First, as a single-center retrospective study, the patient cohort may be subject to selection bias during data collection. In addition, there was no standard protocol for LSA coverage, and the decision was made at the operator’s discretion. Second, the absence of long-term follow-up data is a significant constraint, since patients with trauma are more likely to undergo morphological changes in the aorta as they age, as compared to those with aortic aneurysmal diseases. Therefore, the possibility of additional adverse events occurring over time cannot be ruled out. Third, our retrospective study included only one pediatric patient, a 15-year-old with multiple traumatic injuries from a fall. Given the limited long-term data in the pediatric population, the decision to perform TEVAR was made through multidisciplinary discussion with the trauma team. Further studies are needed to evaluate long-term outcomes in this subgroup. Finally, single-branched devices were not included because they were not available in our region during the study period.

## Conclusion

TEVAR is an effective treatment for BTAI, even in patients with short proximal sealing zones ≤ 15 mm. Early results suggest comparable outcomes of TEVAR between patients with proximal sealing zones ≤ 15 and > 15 mm.

## Data Availability

The data supporting the findings of this study are available from the corresponding author upon reasonable request.
